# The Relationship Internalized Stigmatization and Depression among Adolescents with Substance Use: A Cross-Sectional Study

**DOI:** 10.1192/j.eurpsy.2025.945

**Published:** 2025-08-26

**Authors:** M. Demir, F. C. Gümüş, G. Dikeç

**Affiliations:** 1 Nursing; 2 Psychiatric Nursing, Dicle University, Diyarbakır; 3 Psychiatric Nursing, Fenerbahçe University, İstanbul, Türkiye

## Abstract

**Introduction:**

Although stigmatization is a barrier to treatment for many individuals and groups, individuals with mental disorders are the most affected by stigmatization. Among mental disorders, substance use disorders are the group with high levels of social stigma and internalized stigma. Internalized stigma may lead to a decrease in self-esteem and withdrawal from society and may increase depressive symptoms. Among individuals who use substances, depressive symptoms are high due to both the effects of the substances used and the psychosocial and legal problems experienced. In the literature review, no study examining the relationship between internalized stigma and depression in adolescents was found.

**Objectives:**

This study aimed to examine the relationship between internalized stigma and depression in a group of adolescents diagnosed with substance use disorder.

**Methods:**

The study was conducted between December 2023-March 2024 with 71 adolescents between the ages of 12-18 who were diagnosed with substance use disorder and admitted to the outpatient clinic of university hospital in a city located in the southeastern part of Turkey. Information Form, Internalized Stigma of Mental Disorders Scale-Adolescent Form (ISMI-AF), and Beck Depression Inventory (BDI) were used for data collection.

**Results:**

The mean age of the adolescents was 15.97 years (1.51); 85.9% were male, 59.2% were high school students, and 90.1% had severe depressive symptoms. According to the Pearson correlation analysis, no significant relationship was found between the total scores of the ISMI-AF and BDI; however, BDI, educational status of the adolescent, and academic achievement was found to be statistically significant as determinants of the ISMI-AF (R2=0.29). It was determined that the variables specified in the model explained 24% of the ISMI-AF (Adjusted R Square = 0.24)

**Conclusions:**

Almost all of the adolescents who participated in this study were found to have severe depressive symptoms. In addition to depression, adolescents’ school life and academic achievement were important variables explaining internalized stigma. Since adolescents’ school attendance may be a situation that will prevent substance use, interventions to be carried out in cooperation with mental health professionals and school nurses are very important.

**Disclosure of Interest:**

None Declared

